# Regulating Force in Putting by Using the Borg CR100 scale^®^

**DOI:** 10.3389/fpsyg.2013.00082

**Published:** 2013-02-25

**Authors:** Bo Molander, C.-J. Olsson, Andreas Stenling, Elisabet Borg

**Affiliations:** ^1^Department of Psychology, Umeå UniversityUmeå, Sweden; ^2^Ageing and Living Conditions Programme, Centre for Population Studies, Umeå UniversityUmeå, Sweden; ^3^Department of Psychology, Stockholm UniversityStockholm, Sweden

**Keywords:** putting, subjective force, Borg CR100 scale^®^, psychophysical functions, age, skill

## Abstract

Studies investigating the regulation of force of motor actions are scarce, and particularly so in the area of sports. This is surprising, considering that in most sports precise force is of great importance. The current study demonstrates how a psychophysical scale, the Borg CR100 scale^®^ (Borg and Borg, 2001), can be used to assess subjective force as well as regulate force in putting. Psychophysical functions were calculated on the relationships between judgments of force using the CR100 scale and the length of putting shots, examined in a laboratory setting, where 44 amateur golfers played on both flat and uphill surfaces. High agreement and consistency between CR 100 ratings and distances putted was demonstrated. No significant differences in handling the scale were observed between younger (mean age ≈37 years) and older (mean age ≈69 years) players or between players of different skill level. This study provides a new innovative use of an existing instrument, the Borg CR 100 scale^®^, in order to understand the regulation of force needed for putts of various lengths and surfaces. These results and the potential future benefits of the psychophysical approach in golf are discussed.

## Introduction

In precision sports, such as darts, curling, or golf, the demand of precise force is high in order to achieve good performances. However, despite the importance for athletes to master the amount of force^1^ that needs to be used in a given situation, little is written about how to acquire the necessary skills. In golf, for example, how to make a perfect putt has been evaluated extensively (e.g., Frank, 1999; Pelz, 2002; Utley and Rudy, 2006). However, in this literature very little is said about how to produce the appropriate force of the putt, as compared to how to determine the correct line of the putt. Typically, there is a reliance on the player’s feeling and intuition, and, presumably, a reliance on some unspecified automatically occurring mental and motor processing. Yet, no specific guidelines regarding how to regulate the force of the putts have been offered. Consistent with the above description are results from a study by Beilock and Carr (2001), where intercollegiate golf team members were asked about the steps involved in a typical golf putt. Although one of the steps included looking at the distance of the putt, there was no explicit mentioning of measuring the distance or judging and adjusting the force needed for the putt.

Given the high percentage of putting shots (around 40%) that is reported to be a characteristic of the golf game (Wiren, 1991; Pelz, 2002), the scarcity of concrete advice in the golf instructional literature for how to practice putting force is surprising. Even more surprising is the shortage of scientific studies regarding the regulation of force in tasks in general and in precision sports in particular (see Marchant, 2011, for a review). Scientific studies on force in golf have been directed to issues such as tempo and timing (Jagacinski et al., 2008), perceptual and sensory integration (e.g., Delay et al., 1997; Craig et al., 2000), and video game training (e.g., Fery and Ponsere, 2001). Of most interest in the present context are the studies by Delay et al. (1997) and Craig et al. (2000) on putting, and Jagacinski et al. (2008, 2009) on chipping. These authors suggested that the movement of putting to a specific distance was primarily specified by the magnitude and duration of the backswing. Also, some evidence was obtained for the relative importance of magnitude as compared to duration. However, as many golfers have experienced, adjusting the backswing magnitude, without changing also the acceleration of the club, is difficult and shots will still be too long or too short.

One possibility to extract information about the force needed for a putt is to use a rating scale. Often such scales have 5–9 categories with verbal anchors from, for example, “weak” to “strong,” describing an intensity variation. The advantage of this kind of ordinal category scale is that information of perceived level of intensity is provided. In golf, the presence and use of verbal descriptors could make it easier and more natural for participants to determine the force required for a putt, by focusing on the subjective force, and to improve communication about a shot performed (cf. Landin, 1994).

The verbal descriptors can be seen as summaries of the experiences individuals have about the subjective force needed for striking, pushing, and moving objects which can be seen as tools easier to use during the production of putting shots than just referring to feeling or intuition. Usually, ordinal category scales are rather rough, and since these scales are ordinal the use of parametric statistics is questionable. For the purpose of this study something more fine-graded and statistically powerful is needed.

**Figure 1:**
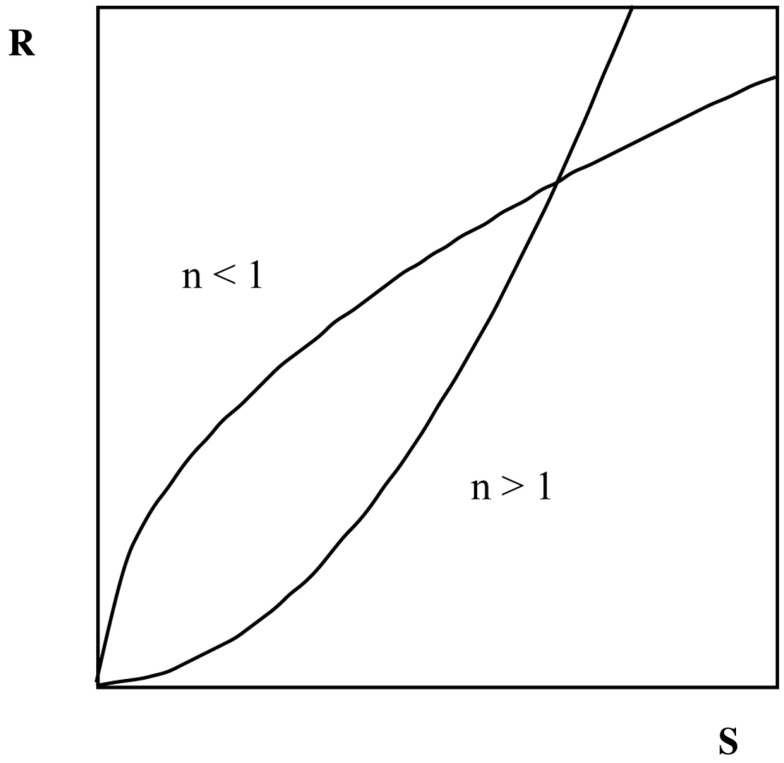
**Two theoretical S-R functions obtained with ratio scaling**. Examples of exponents above 1 (*n* > 1) are *perceived exertion* (1.6), *heaviness* (1.45), *muscle force* (1.7), and *electric shock* (3.5). Examples of exponents below 1 (*n* < 1) are *brightness* (0.3–0.5), *loudness* (0.6), and *visual area* (0.7).

One option is to use the psychophysical ratio scaling methods developed by Stevens (1957). Using the method of magnitude *estimation*, participants utilize numbers without restrictions to match the perceived intensities of perceptions or feelings. In magnitude *production*, participants instead produce the physical intensities to match preset subjective magnitudes. A great advantage of these methods is the possibility to calculate psychophysical power functions, describing relative growth of subjective or perceived intensity with physical intensity. As originally shown by Stevens (1957): *R* = *c* × *S^n^*, where *R* is the perceived intensity, *c* is a measure constant, *S* is the physical intensity, and *n* is the exponent describing the curvature of the function. As illustrated in Figure 1 an exponent below *n* = 1 describes a negatively accelerating function where an equal stimulus increase is perceived as a successively smaller perceptual increase, and an exponent above *n* = 1 describes positively accelerating functions where an equal stimulus increase is perceived as a successively larger perceptual increase.

There is extensive literature showing the usefulness of psychophysical scaling in various areas (e.g., Borg, 1962, 1998; Carroll and Arabie, 1980; Young, 1984; Gescheider, 1988, 1997; Algom, 1992). Numerous applications have appeared also in sports, often for determining and regulating exertion and effort (e.g., Borg, 1986; Mori, 1999; Tenenbaum, 2001). Some disadvantages with the methods are, however, that they lack the inherent possibilities of direct inter-individual comparisons and level determinations. Therefore, G. Borg developed the Borg CR (Category-Ratio) scales that combine the advantages of Stevens’ ratio scaling for description of psychophysical functions with the advantages of category scaling (Borg, 1986; Borg and Borg, 2002).

**Figure 2:**
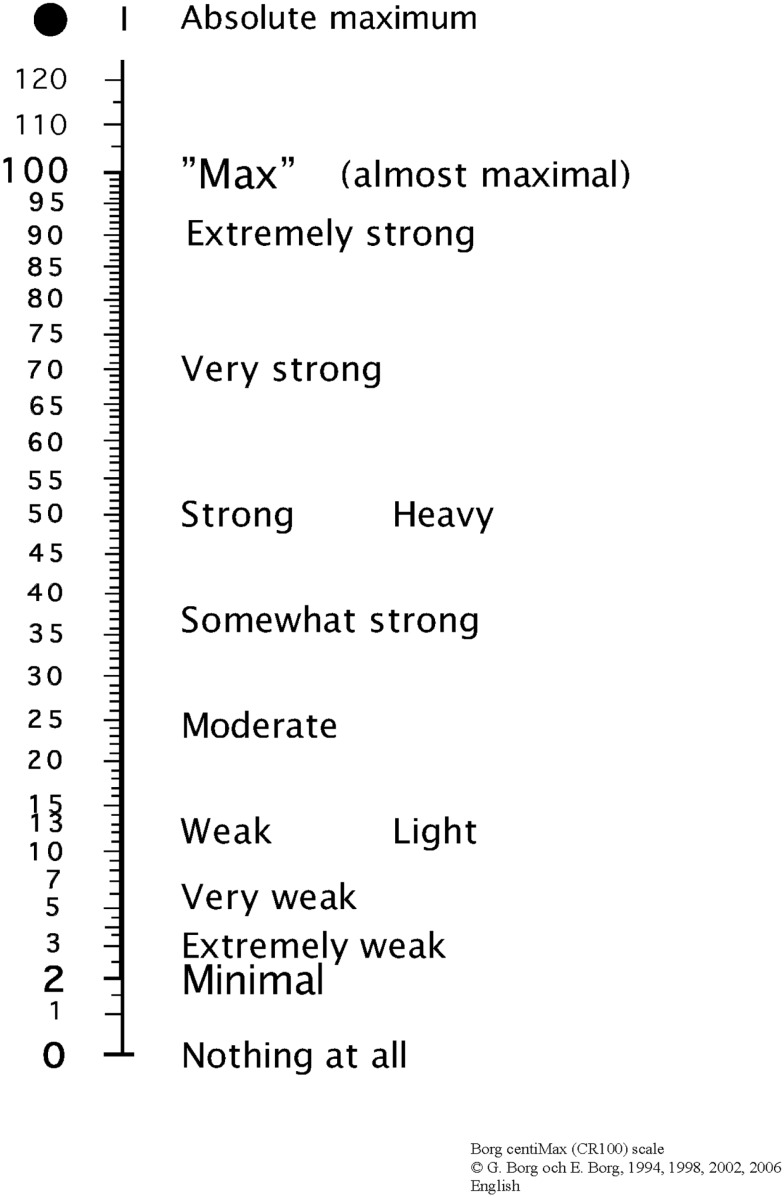
**The Borg CR100 scale^®^**. For users of the scale the latest version and instructions are provided by borgperception@telia.com.

In the present study we examine the value of such a scale during putting using golfers at different levels of skill and age. In particular, we examine how subjective force, rated with the scale, is influenced by a flat or uphill putting surface. The scale used here is the Borg CR100 scale^®^, sometimes called the *centiMax* scale, as it measures intensity in centigrade of a maximal experience. This scale varies from 0 to 100 (see Figure 2), with the verbal descriptors placed where they belong on a ratio scale, i.e., so as to give ratio data comparable to what is obtained with traditional psychophysical methods such as magnitude estimation (Borg and Borg, 2001; Borg and Kaijser, 2006; Borg, 2007). Thus, 0 is described as “nothing at all” (i.e., no subjective force), and 100 is described as *maximum* (i.e., maximal subjective force) and anchored in a previous experience of a maximal perceived exertion. In between these points the descriptors *minimal*, *extremely weak*, *very weak*, *weak*, *moderate*, *somewhat strong*, *strong*, *very strong*, and *extremely strong* are interspersed. The scale also allows for *absolute maximum*, a value above 100, which can be given by the participant in the rare occasions he/she has to produce or perceives a magnitude of force never needed or experienced before.

Presumably, the use of verbal descriptors will make it easier and more natural for participants to determine the intensity needed for a putt. Verbal descriptors will thus contribute to better inter-individual agreement about how to use the scale (e.g., Gescheider, 1997). Even novices, who have limited experience of putting, have a vast experience of using force in everyday life and are therefore capable of matching force to expressions such as *weak* and *very weak*. Thus, novices and skilled golfers alike are capable of using quantitative scales. At the same time, by its many steps, the Borg CR100 scale^®^ allows the player to fine-grade the force of the putting shots during training and practice. Furthermore, the ratio properties of the scale enable descriptions of psychophysical growth functions similar to what is obtained with, for example, magnitude estimation (cf. Figure 1).

In the present study “force” for the muscular effort of regulating the putt is used in the sense of “subjective force” or “perceived force.” This specific meaning of force was made clear to the participants in the instructions of how to use and understand the CR100 scale. However, it should be noted that subjective or perceived force may be regarded as a “gestalt,” integrating information from several modalities and perceptual systems, much in a similar way as with overall perceived exertion (Borg, 1998; see also. Jones, 1986; Gescheider et al., 1992; Jagacinski et al., 2009).

In a preliminary study (Molander et al., 2005) the Borg CR100 scale^®^ was used in putting in a laboratory setting as well as on a golf course green. Of interest here are data from 12 golf players, who participated in both conditions, and data from seven young elite players recruited from an elite program, who participated only in the field condition. The mean age of these players was 22.8 years (range 15–33 years) and the mean golf handicap was 12.3 (range 2–28). Thus, these players were moderately skilled. In both field and laboratory settings putting was performed under two different instructions, that is, *production* and *estimation*. In the *production* procedure the player produced putts of a chosen subjective force as specified in the CR100 scale (e.g., weak, moderate, strong), and in the *estimation* procedure the player made putts at different distances to a hole and then estimated the force used according to the CR100 scale. None of the participants had used a psychophysical scale before.

**Figure 3:**
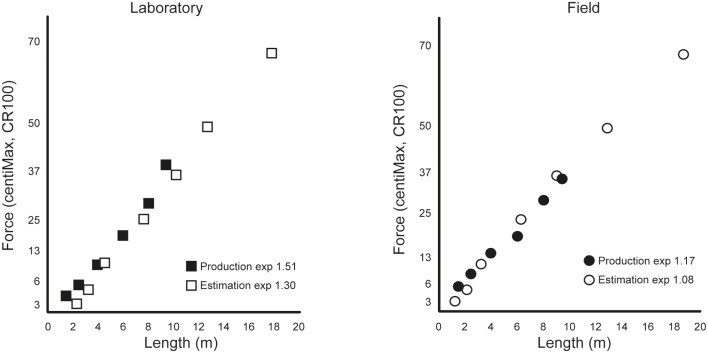
**Psychophysical relationships between CR100 intensity and putting length (m) obtained in the laboratory and field part of the preliminary study**.

Figure 3 shows plots of how the players succeeded to produce putting distances to given scale values (*production*) and to relate scale values to the distance of the putting shots (*estimation*). *Production* and *estimation* procedures give very similar growth functions, although *estimation* exponents are often somewhat lower than *production* exponents. High similarity between *production* and *estimation* functions provides evidence for the validity of the scale (cf. Gescheider, 1997) and demonstrates that participants are using the scale in a consistent manner. The similarity between the laboratory and field plots in Figure 3 are high, in view of (1) the big difference in ball speed in the two settings, the field course being much slower than the laboratory course, and (2) the lack of experience of playing on fast greens for most of the participants. Also, these data demonstrate that the scale worked well for short, as well as long, distances. The difference in exponents between conditions can be seen as proof of the sensitivity of exponents for changes in the context due, for example, to such factors as differences in experience in playing on field greens, or differences in perception of the two different putting conditions. Taken together the participants produced stable psychophysical functions and were able to use the CR100 scale properly in both laboratory and field conditions.

However, two shortcomings of the preliminary study justify further evaluation of the use of psychophysical scaling in putting. First, the instructions for *production* deviated somewhat from what we believe golfers are actually doing when putting during realistic field conditions. This deviation is corrected in the present study. Second, as the preliminary study was predominantly performed on flat surfaces there is need to study the use of the CR100 scale also on a sloped surface. Carnahan (2002) suggested that putting upwards is easier than other putts, this is further examined in the present study but in a controlled laboratory setting. Moreover, it is also examined whether there is any difference of the shape of the psychophysical function between flat and uphill conditions. Also, all players will play on both flat and uphill surfaces, which give us an opportunity to study if change of conditions (flat vs. uphill and vice versa) affects judgments of force and putting precision. Finally, the study comprises participants over a larger age span and players with longer experience of golf than the participants in the Molander et al. (2005) study. How the oldest players handle the psychophysical scale is of particular interest, as this group might be reluctant to change old habits, or likely to experience the psychophysical procedure as cognitively demanding (e.g., Tun and Wingfield, 1995; Pesce et al., 2007).

Summarizing, the main purpose of this study is (1) to demonstrate that the Borg CR100 scale^®^ is an easy-to-use tool for regulating force in putting in younger and older players. The purpose is (2) also to show how psychophysical functions can add important information about the putting behavior of the players in an experimental setting.

Specifically, we examine (a) psychophysical functions as obtained in putting on flat and uphill surfaces and under instructions of *production* and *estimation* procedures. It is expected that *production* and *estimation* will yield highly similar functions in flat and uphill conditions, respectively. Also examined are (b) psychophysical functions (i.e., *production*) and putting performance when changing from flat to uphill surface and from uphill to flat surface. Based on findings from a previous study (Carnahan, 2002) we expect putting to be better (i.e., closer to target) on uphill as compared to flat surface. Thus, it is reasonable to expect a difference in size of exponents between uphill and flat psychophysical functions. Finally, we examine (c) psychophysical functions and putting performance in younger and older players, and among players with different skill levels. If the older players suffer from cognitive and motor deficits to a greater extent than the younger players, as the cognitive aging literature suggests, differences between these two groups with respect to exponents should be expected.

## Materials and Methods

### Participants

In this study 44 players participated, one woman and 43 men, with a mean age of 56.0 years (range 21–80 years). The mean handicap was 13.8 (range 4–20) and the mean experience of playing golf was 18.1 years (range 4–55 years). The mean competitive golf experience was 13.0 years (range 0–30 years). Most of the participants were members of local clubs and the experimenter recruited all participants. Requirement for being recruited to the study was that the player’s handicap was 20 or below. All players played from the right side. Informed consent was obtained for participation in the study. The players spent about 1 h in the laboratory and received three golf balls and a green tool to a value of approximately 20 US$ for their participation.

### Apparatus

**Figure 4:**
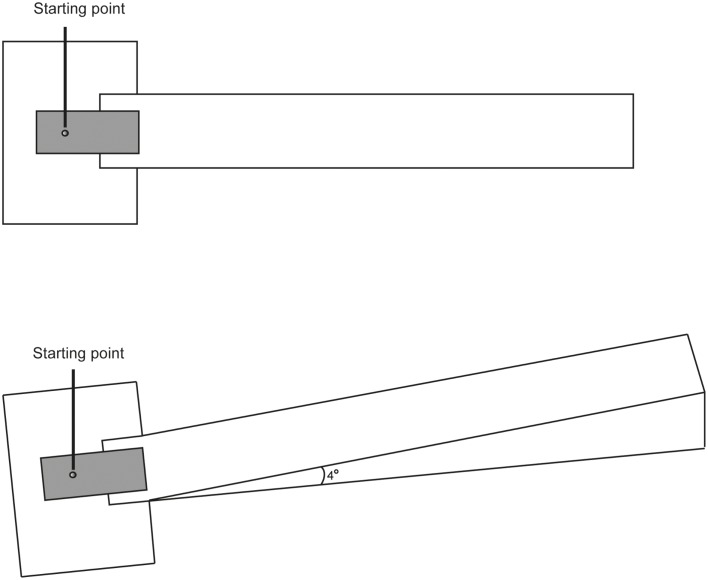
**Laboratory setup**. The uphill surface had an angle of inclination of 4°.

The study took place indoor in a laboratory equipped with two miniature-golf courses on which putting was performed. One course was a flat 11 m long wooden construction (see Figure 4), and the other was a 10.7 m long uphill course, also a wooden construction, with an angle of inclination of (4°). Both constructions had the same width (0.9 m) and height of border (0.04 m). Both courses were covered with a special felt surface (Astro Turf), simulating the surface of a grass green. The ball rolled 3.50 m (≈11.5 feet) on the textile surface as measured by a stimpmeter (KSAB Golf Equipment, Sweden). Thus, the speed on the surface was close to what Pelz (2002, p. 328) denotes as “PGA-Tour fast.” Players used the same club (Greenmaster Professional Edge GM-04), and the same ball (Titleist XL). A measuring tape placed along the border of the courses measured the length of each putt. The players were not able to see the measuring tape during the putting.

### Instrument

The Borg CR100 scale^®^ (Borg and Borg, 2001; Borg, 2007) was used for ratings of subjective force (see Figure 2). Generally, psychophysical scales are thoroughly secured through the procedure of construction, definitions, and instructions for use of the scale (cf. Neely, 1995). Thus, validity and reliability can be expected to be high. Advantages of CR100, as compared to the Absolute Magnitude Estimation scale (e.g., Zwislocki and Goodman, 1980), are shown by Borg and Borg (1998) and Borg (2007).

### Procedure

Participants were recruited by telephone and asked about background information, such as age, handicap, and golf experience. Based on these data participants were placed in two groups. One group (AB) started putting on the uphill course and then changed to the flat course, and one group (BA) started putting on the flat course and then changed to the uphill course. The two groups were matched by age and handicap. The participants were studied individually. Upon arrival to the laboratory the rating skill of the participant was tested by using materials for judging shades of black consisting of 5 cm × 5 cm fitted on A4-sized sheets of paper and varying in degree of blackness. Ratings of blackness were done with a scale similar to the CR100 scale. The purpose of this test was to ensure that the participants would understand, as well as become familiarized with, the CR100 scale. All participants passed the test without problem. Then, background information was again recorded, including age, handicap score, number of years as a golfer, number of years of competitive experience, and preferred side of playing. Participants were given detailed instructions on how to use the scale. Below is the standard introductory part of the instructions: “Use this rating scale to tell how strong your experience is. Your experience can be about effort, pain, or something else. One hundred (“Maximum”) is a very important level of intensity. This level is to be considered as a referential point on the scale. It is the strongest experience or feeling (for example, from effort) you ever had. Still, it is possible to experience or imagine something even stronger. For that reason there is on the scale “Absolute maximum” at the top of the scale, marked with a “•”. If your experience or feeling is stronger than 100, you can use a higher number. Usually the intensity in our experiences is not as strong as 100, and often numbers below 2–3 are not needed either. The scale works as a percentage scale, and there is very good agreement between what the verbal expressions means and what the numbers stand for” (cf. Borg, 1998).

In addition, elaborations of the introduction were presented, exemplifying putting situations and ensuring that participants were aware of the correspondence of the verbal and numerical expressions. Also, it was emphasized that any number on the scale could be used, including numbers without verbal expressions. The player was then given 5 min of practice to accommodate to the club, ball, and the speed of the ball on the surface. Practicing took place both on the flat and the uphill course. The CR100 scale was put down on the floor beside the player, visible for use. After finishing the practice period the player started the experiment, which consisted of four phases. Table 1 gives an overview of the four phases for each of the two groups of players.

**Table 1:** **Experimental phases for Group AB and Group BA**.

Group AB	Phase 1 (uphill)	Phase 2 (uphill)	Phase 3 (uphill)	Phase 4 (flat)
	Production	Estimation	Production	Production
Group BA	Phase 1 (flat)	Phase 2 (flat)	Phase 3 (flat)	Phase 4 (uphill)
	Production	Estimation	Production	Production

*Each phase consisted of six target distances. Participants putted twice at each target distance within phases in random order. In total participants putted 12 putts per phase*.

Phase 1 was the *production* phase. A circular cardboard, simulating a hole on a regular green, was put out on the surface in six different distances in random order. The task of the player was to make a putt as close as possible to the hole. The hole on the cardboard was shaded in black to create an illusion of a real hole. The golf ball could pass over the hole without changing much speed or direction. Before making the putt the player rated the force believed to be necessary and sufficient to reach the hole by using the CR100 scale. If needed, the player was allowed to use any number in between the verbal expressions. The task was then to produce the envisioned subjective force chosen from the CR100 scale. The six given target distances were 1.5, 2, 3, 4, 6, and 8 m. All distances were presented twice.

Phase 2 was *estimation*. Here, the player attempted to putt as close as possible to the various distances marked by the cardboard hole. After each putt the player rated the force of the putt using the CR100 scale. In this phase there were also six, slightly different, distances: 1, 2.75, 3, 5, 7, and 8.5 m. These distances were also randomly presented twice. The difference in distances between phases 1 and 2 was due to the purpose of examining different distances.

Phase 3 and 4 followed both the *production* protocol. Phase 3 was a repetition of the *production* procedure in phase 1. In phase 4, the player moved to the other course. In this phase, putts were made according to the *production* procedure and the target distances in phases 1 and 3, again with the distances being presented twice. In all phases the ratings and the distances of all putting shots were recorded.

### Statistical analyses

**Figure 5:**
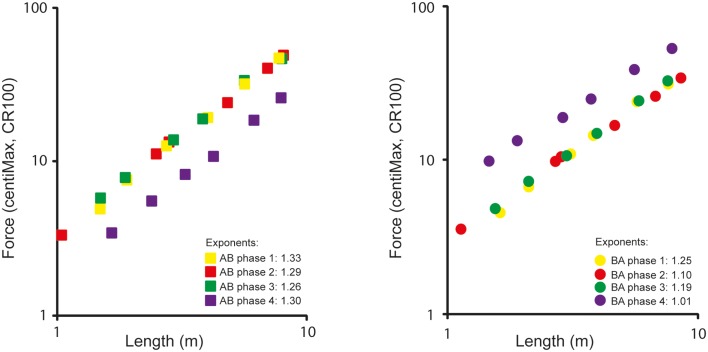
**Psychophysical relationships between CR100 intensity and putting length (log m) for AB and BA groups during phases 1 to 4**. Note. In accordance with psychophysical customs, and in order to ease comparisons, the power functions in this figure are expressed as linear “log–log plots.”

Psychophysical power functions were calculated according to Stevens (1957) equation (*R* = *c* × *S*^n^), as described above, in order to study the relationship between distance (S) and perceived force (*R*). When taking the logarithm of the power function (log *R* = log *c* + *n* × log *S*), the exponent is obtained as the slope in a linear regression equation (thus, a linear relationship in log–log coordinates), and the measure constant (c) corresponds to the intercept. Power functions shown in Figure 5 are expressed as linear logarithmic functions (log–log).

Mixed analyses of variance (ANOVAs) were performed on putting data, rating data, and exponents in order to illuminate any differences between groups, phases, and distances. To study the difference between a flat and uphill surface, mixed 2 (group) × 2 (phase) × 6 (distance) ANOVAs were performed on the *production* putting distances data and rating data (phase 1 and 4). Phase and distance were the within-subject factors and group was the between-subject factor. Separate ANOVAs were performed on putting data and rating data. Mixed 2(group) × 2(skill) × 2(phase) × 3(distance) ANOVAs were performed on putting performance and ratings in phase 1 and 4, and at target distances of 1–2, 3–4, and 6–8 m in order to study the effect of skill. Phase and distance was the within-subject factors, while group and skill were the between-subject factors. Also here were separate ANOVAs performed on putting data and rating data. In addition, a mixed 2 (group) × 2 (phase) ANOVA was performed to illuminate any differences in exponents, with phase as the within-subject factor and group as the between-subject factor (phase 1 and 4). The level of significance was set to 0.05, and effect sizes were calculated using partial Eta squared ($\eta_{p}^{2}$). Bonferroni adjustment was applied in *post hoc* analyses. Correlations were Pearson correlations, with *z*-test used for testing the difference between correlations (Bruning and Kintz, 1977). Correlations were calculated for the log–log relationships between distances of the putting shots and the corresponding CR100 scale rating values.

Median splits were made in each of the two experimental groups for studying effects of age and skill. In the AB group younger players had a mean age of 37.5 years (*N* = 9, range 22–59 years) and the older players had a mean age of 69 years (*N* = 13, range 65–80 years). In the BA group the corresponding mean ages were 36.3 years (*N* = 9, range 21–54 years) and 69.4 years (*N* = 13, range 65–80 years). For skill, the two experimental groups were split according to handicap under and above 14. In Group AB the handicap means were 10.5 (*N* = 12, range 4.7–13.0) and 18.0 (*N* = 10, range 15.3–20.2) for the subgroups under and above 14, respectively, and for BA the corresponding means were 9.7 (*N* = 11, range 4.0–13.4) and 17.7 (*N* = 11, range 14.4–20.1).

IBM SPSS Statistics (PASW) v.18 was used for the statistical calculations.

## Results

In this section we present results that (a) demonstrate characteristics of the obtained psychophysical functions in putting, (b) compare the putting and psychophysical performance of the two groups (AB and BA), when putting on the uphill surface and flat surface, focusing also on changes in performance when players move from putting on uphill to flat surface and from flat to uphill surface. Finally, we will (c) examine the effects of age and skill on the use of the CR100 scale in putting.

### Psychophysical functions: Production and estimation

Obtained psychophysical functions are shown in Figure 5. Functions are shown as log–log plots and presented separately for the AB and BA players. The mean *production* exponent, calculated over all players in the first *production* phase, was 1.29. Overall, the mean *estimation* exponent was 1.21. This difference was significant (*p* = 0.01). It should be noted in Figure 5 that with increased practice over the first three phases on the same course the exponents tend to decrease nominally. Thus, for both the flat and the uphill course the size of the *production* exponent decreases, and, overall, from 1.29 in phase 1 to 1.23 in phase 3. However, this decrease did not reach an acceptable level of significance (*p* > 0.05).

### Effects of flat vs. uphill course

Results showed that there was a significant difference between Group AB (starting uphill) and Group BA (starting flat) (*F*_1,42_ = 9.71, *p* < 0.01, $\eta_{p}^{2}=0.19$). Overall, Group BA putted slightly closer to the different holes than Group AB. There was also a significant group × phase interaction effect (*F*_1,42_ = 21.9, *p* < 0.01, $\eta_{p}^{2}=0.34$), and a significant group × phase × distance interaction effect (*F*_2.79,117.3_ = 4.60, *p* < 0.01, $\eta_{p}^{2}=0.10$), the latter interaction being Greenhouse–Geisser corrected because of violation of the sphericity assumption. The first interaction shows that in Group AB, mean putting lengths increase when changing from uphill to flat course, whereas in Group BA, mean putting lengths shorten when changing from flat to uphill course. Inspection of the second interaction indicates that, for AB after change, putting length increases with the increasing distance to putt, whereas for BA there is no trend over increasing target distances.

For rating values there was a significant effect of group (*F*_1,42_ = 7.01, *p* < 0.01, $\eta_{p}^{2}=0.14$). Group AB used lower values of the CR100 scale than Group BA. There were also effects of group × phase (*F*_1,42_ = 116.90, *p* < 0.01, $\eta_{p}^{2}=0.74$), and group × phase × distance (*F*_1.67,42_ = 57.6, *p* < 0.01, $\eta_{p}^{2}=0.58$). These interactions showed that Group AB decreased rating values when changing to flat course, whereas Group BA increased rating values when changing to uphill course. Ratings in the two groups followed a parallel course over distances in the slope condition, whereas differences between the groups increased with distance in the flat condition. The 2(group) × 2(phase) ANOVA on exponents showed only an effect of phase (*F*_1,42_ = 11.36, *p* < 0.01, $\eta_{p}^{2}=0.21$), indicating that the *production* exponents were higher in the first phase than the fourth phase.

Over the two groups, the correlation for each of the phases was 0.98, 0.98, 0.98, and 0.77 for phase 1, phase 2, phase 3, and phase 4, respectively. The decrease in the fourth phase was due to lower correlations for both of the groups, Group AB showing a coefficient of 0.75 and Group BA a coefficient of 0.84. All correlations were significant (*p*’s < 0.05). The difference between 0.75 and 0.84 in phase 4 was tested by the Bruning and Kintz (1977) *z*-test for difference between independent correlations (*p* < 0.01). The *z*-test difference result indicates a larger discrepancy between putting distances and rating values in the group AB, who changed from uphill to flat course, compared to group BA, who changed from flat to uphill course.

### The effect of age

For exponents obtained in phase 1 under *production* instructions, *t*-tests showed no significant differences between younger and older players in any of the AB and BA groups (*p*s > 0.05). Similar tests in phase 1 for putting distances and rating values also showed no significant age differences (*p*s > 0.05).

### The effect of skill

There were no significant main effects of skill; neither was there any significant interaction involving skill. For the main effect of skill on ratings, the analysis showed *F* < 1, $\eta_{p}^{2}=0.01,$ and for the main effect of skill on putting, the analysis showed *F* < 1, $\eta_{p}^{2}=0.02.$

Consistent with these results were correlations between ratings and putting performances calculated in each skill group and over all conditions. For players with handicap under 14 and over 14 these correlations were 0.82 and 0.76, respectively. The difference between these coefficients was not significant (*p* > 0.05), suggesting that skill level does not affect the use of the scale.

## Discussion

The main aims of the current study were (1) to demonstrate that the Borg CR100 scale^®^ is an easy-to-use tool for regulating force in putting, and (2) to show how psychophysical functions as studied in an experimental setting can contribute important information about the putting behavior of the players.

### The CR100 scale as a tool to improve putting performance

Considering the fact that none of the players had any previous experience of the CR100 scale, as well as no previous experience of the fast “indoor surface,” the results of this study showed consistent functions of the relationships between putting distances and scale ratings. Exponents of the functions were all above 1.0, with the exponent of 1.25 in the first *production* phase on the flat surface as a suitable reference for the other phases and conditions. In comparison, Gescheider (1988, 1997) reported exponents for rated length of lines (1.0), pressure on palm (1.10), heaviness (1.45), and muscle force (1.70). Thus, the values obtained in the present study suggest that subjective ratings based on putting differ from subjective ratings based on just distances or muscles. A possible modality of relevance for putting is perceived speed, e.g., speed of the ball or speed of the downswing. Psychophysical studies of speed are, however, difficult to find. In a study by Stevens (1975) acceleration was studied and the reported exponent was 1.40. It is of interest to discuss possible types of exponents, as well as their sizes, because instructions and procedures for learning how to putt should be dependent on the modalities necessary for the task. The current study contributes to such basic knowledge. The exponents from the present study represent values reflecting that the Borg scale is easy to learn and a promising instrument for regulating subjective force in putting, if this also improves putting performance is for future studies to examine. To our knowledge, this study, including the preliminary results referred to in the introduction section, is the first attempt to determine the psychophysical functions in putting. The psychophysical functions for putting force under the conditions of the present study were positively accelerating indicating that at short distances the perceived needed force increased more slowly. However, at long distances the perceived needed force increased more rapidly. This implies that when adding the same amount to the distance, the amount of force needed to be added to produce that extra distance becomes larger and larger (cf. Figure 1). Factors, such as speed of the green and slope of the green, may of course modify functions and size of exponents.

### Effects of flat vs. uphill course

The variation of situations of force judgment in golf is large and often occurring in complicated circumstances, but we know very little of the functions involved. It would also be interesting to compare the exponents of putting with exponents obtained in other types of shots in the golf game, such as chipping and bunker shots. As this study shows, the exponent is sensitive to changes in conditions. For example, the exponent increased from flat to uphill course and it was reduced when repeating a phase (compare phase 1 to phase 3). The ANOVA analyses of putting shots and ratings showed a Group by Phase interaction, such that Group AB increased the distances of the shots by going from uphill to the flat course, whereas Group BA decreased the distances by going from the flat to the uphill course. These changes were more pronounced for the AB group than for the BA group. Another illustration of the effect is the correlation pattern between shot distances and ratings. From high values of around 0.98 in the three first phases, Group AB and Group BA decreased the correlation coefficients to 0.75 and 0.84, respectively, in phase 4. It is important from a practical point of view that players are aware of such an effect, which seems to be more pronounced when going from uphill to flat course than vice versa. Unless shots and ratings are recorded, as in the present study, this mismatch between the motor system and rated force, presumably giving rise to negative transfer, probably goes unnoticed during ordinary games. Studying such recordings is likely to give valuable advice for putting practice.

Moreover, the results point to a slight overall advantage for the play on a flat surface. The players that started on the flat course (BA group) overestimated shots in shorter target distances and underestimated shots in longer target distances. The players that started with the uphill course (AB group) underestimated shots in all target distances. For both groups playing uphill the underestimation tended to increase with increased target distance, underpinning the conclusion reached by Pelz (2002), that a sloped surface gives the visual impression that the target is closer to the player than it actually is, producing underestimation of the subjective force of the putt. However, the present results are contradictive to the results by the Carnahan (2002) study showing that uphill putts are easier than other putts. Consequently, the generalizability of Carnahan’s (2002) results can be questioned. It should be noted, though, that one important difference between the Carnahan study and the present one is that in the former study the task was to hole the putt. In this study the task was to make the shot distance as close as possible to the target distance. Moreover, although there are some advantages to study putting in field conditions, some questions, such as the present one, are better examined under controlled laboratory conditions. For example, putting uphill in the field is not only dependent on the uphill angle; there are also effects of other factors, such as breaks in varying directions or surfaces with varying ball speeds.

### The effect of age and skill

Previous studies have shown that during competitive high-arousal conditions motor performance, as well as cognitive performance may be impaired in golf players in general, and to a greater extent in older players than in younger players (e.g., Molander and Bäckman, 1996; Beilock et al., 2002). This is further supported by studies showing cognitive and motor deficits and impairments in the elderly (e.g., Schaie and Willis, 2011). In the present study no differences were found between the younger and older players, neither for using the scale nor for putting performance. Hence, across different ages, the CR 100 scale appears to function properly during putting. One important aspect of the younger-older question is that the study was performed in a non-competitive atmosphere, the participant being alone with the experimenter, working at his own pace. Thus, potential stress associated with competition was reduced. Finally, our results illustrate that the handicap point is not a good indicator of putting skill, also supported by Carnahan (2002), at least not for the handicap range that was present here. Further, the pattern of the CR100 scale ratings convincingly suggests that the difference in judging subjective force between low- and high-handicap players generally increases with increasing distance to the target. This is information that helps us to better understand the motor-cognitive relationships.

## Conclusion and Future Directions

The present study shows that golfers of different age, golf handicap, and competitive experience in ranges common for amateur golfers easily adapt to the instructions for using the Borg CR100 scale^®^ in a putting situation. What the CR100 scale is likely to add is to provide a stable structure and routine for regulating the force of the putt in the same way from shot to shot and from green to green. Given that individual functions are calculated, tools for predicting the force needed for distances not played before can be provided. Finally, this study illustrates several examples of how a scale such as the CR100 scale can be of benefit in experimental settings, where relationships between motor and cognitive processes are studied. It would be interesting for future studies to examine how the CR 100 scale could be used in putting practice, and whether the use of a scale like the CR 100 actually improves putting performance. If putting distances and ratings in practice would to be registered, players and coaches could potentially learn more of the individual variation as well as how to regulate force for putting during different conditions. However, this remains to be tested. Another interesting question for future studies is whether the support of a scale such as the CR100 may actually reduce some of the tensions and cognitive mistakes observed in younger and older players, provided the use of the scale is well learned.

## Conflict of Interest Statement

The authors declare that the research was conducted in the absence of any commercial or financial relationships that could be construed as a potential conflict of interest.

## Footnotes

^1^In the context of the present paper, the meaning of “force,” or more properly, subjective or perceived force, is reflecting a predominantly perceived muscular effort.

## References

[B1] Algom D. (ed.). (1992). Psychophysical Approaches to Cognition. London: North-Holland

[B2] Beilock S. L. Carr T. H. (2001). On the fragility of skilled performance: what governs choking under pressure? J. Exp. Psychol. Gen. 130, 701–725 10.1037/0096-3445.130.4.701 11757876

[B3] Beilock S. L. Carr T. H. MacMahon C. Starkes J. L. (2002). When paying attention becomes counterproductive: impact of divided versus skill-focused attention on novice and experienced performance of sensorimotor skills. J. Exp. Psychol. Appl. 8, 6–16 10.1037/1076-898X.8.1.6 12009178

[B4] Borg E. (2007). On the Perceived Exertion and its Measurement. Ph.D. thesis, Stockholm University, Stockholm

[B5] Borg E. Borg G. (1998). “Absolute ME compared with Natural CR scaling,” in Fechner Day 98. Proceedings of the Fourteenth Annual Meeting of the International Society for Psychophysics, eds Grondin S. Lacouture Y. (Quebec: The International Society for Psychophysics), 199–204

[B6] Borg E. Borg G. (2002). A comparison of AME and CR100 for scaling perceived exertion. Acta Psychol. (Amst.) 109, 157–175 10.1016/S0001-6918(01)00055-5 11820425

[B7] Borg E. Kaijser L. (2006). A comparison between three rating scales for perceived exertion and two different work tests. Scand. J. Med. Sci. Sports 16, 57–69 10.1111/j.1600-0838.2005.00448.x 16430682

[B8] Borg G. (1962). Physical Performance and Perceived Exertion (Doctoral thesis). Studia Psychologica et Paedagogica: Series Altera, Investigationes XI. Lund: Gleerup.

[B9] Borg G. (1986). “Some studies of perceived exertion in sports” in The Perception of Exertion in Physical Work, eds Borg G. Ottoson D. (London: MacMillan), 293–302

[B10] Borg G. (1998). Borg’s Perceived Excertion and Pain Scales. Champaign, IL: Human Kinetics

[B11] Borg G. Borg E. (2001). A new generation of scaling methods: level-anchored ratio scaling. Psychologica 28, 15–45

[B12] Bruning J. L. Kintz K. L. (1977). Computational Handbook of Statistics, 2nd Edn Abingdon: Scott, Foresman and Company, 214–215

[B13] Carnahan J. V. (2002). “Experimental study of effects of distance, slope and break on putting performance for active golfers” in Science and Golf IV. Proceedings of the World Scientific Congress of Golf, ed. Thain E. (London: Routledge), 113–126

[B14] Carroll J. D. Arabie P. (1980). Multidimensional scaling. Annu. Rev. Psychol. 31, 607–649 10.1146/annurev.ps.31.020180.003135 20812811

[B15] Craig C. M. Delay D. Grealy M. A. Lee D. N. (2000). Guiding the swing in golf putting. Nature 405, 295–296 10.1038/35012690 10830947

[B16] Delay D. Nougier V. Orliaguet J.-P. Coello Y. (1997). Movement control in golf putting. Hum. Mov. Sci. 16, 597–619 10.1016/S0167-9457(97)00008-0

[B17] Fery Y.-A. Ponsere S. (2001). Enhancing the control of force in putting by video game training. Ergonomics 44, 1025–1037 10.1080/00140130110084773 11780726

[B18] Frank J. (1999). Precision Putting. Champaign, IL: Human Kinetics

[B19] Gescheider G. A. (1988). Psychophysical scaling. Annu. Rev. Psychol. 39, 169–200 10.1146/annurev.ps.39.020188.001125 3278675

[B20] Gescheider G. A. (1997). Psychophysics. The Fundamentals, 3rd Edn London: LEA

[B21] Gescheider G. A. Bolanowski S. J. Jr. Verrillo R. T. (1992). “Sensory, cognitive and response factors in the judgement of sensory magnitude” in Psychophysical Approaches to Cognition, ed. Algom D. (London: North-Holland), 575–622

[B22] Jagacinski R. J. Kim T. H. Lavender S. L. (2008). “Rythmic force patterns in the golf swing” in Science and Golf V. Proceedings of the Scientific Congress of Golf, eds Crews D. Lutz R. (Tempe, AZ: World Scientific Congress of Golf Trust), 82–88

[B23] Jagacinski R. J. Kim T. H. Lavender S. L. (2009). Managing the rhythmic complexity of hitting a golf ball. J. Mot. Behav. 41, 469–477 10.3200/35-08-075 19508955

[B24] Jones L. A. (1986). Perception of force and weight: theory and research. Psychol. Bull. 100 29–42 10.1037/0033-2909.100.1.29 2942958

[B25] Landin D. (1994). The role of verbal cues in skill learning. Quest 46, 299–313 10.1080/00336297.1994.10484128

[B26] Marchant D. C. (2011). Attentional focusing instructions and force production. Front. Psychol. 1:210 10.3389/fpsyg.2010.00210 21833266 PMC3153816

[B27] Molander B. Bäckman L. (1996). Cognitive aging in a precision sport context. Eur. Psychol. 1, 166–179 10.1027/1016-9040.1.3.166

[B28] Molander B. Borg G. Olsson C.-J. (2005). “Natural scaling in golf putting” in Science and Profession. Challenge for the Future. Proceedings of the 4th International Scientific Conference of Kinesiologoy, eds Milanovic D. Prot F. (Zagreb: University of Zagreb), 703–706

[B29] Mori S. (1999). “Toward a study of sports psychophysics,” in Fechner Day 99: The End of 20th Century Psychophysics. Proceedings of the 15th Annual Meeting of the International Society for Psychophysics, eds Killeen P. R. Uttal W. R. (Tempe, AZ: Arizona State University), 308–313

[B30] Neely G. W. (1995). Category-Ratio Scaling of Sensory Magnitude in Comparison with Other Methods. Ph.D. thesis, Stockholm University, Edsbruk

[B31] Pelz D. (2002). Dave Pelz’s Putting Bible. “The Complete Guide to Mastering the Green.” New York: Random House

[B32] Pesce C. Cereatti L. Casella R. Baldari C. Capranica L. (2007). Preservation of visual attention in older expert orienteers at rest and under physical effort. J. Sport Exerc. Psychol. 29, 78–99 17556777 10.1123/jsep.29.1.78

[B33] Schaie K. W. Willis S. L. (eds). (2011). Handbook of the Psychology of Aging, 7th Edn San Diego: Academic Press

[B34] Stevens S. S. (1957). On the psychophysical law. Psychol. Rev. 64, 153–181 10.1037/h0046162 13441853

[B35] Stevens S. S. (1975). Psychophysics: Introduction to its Perceptual, Neural, and Social Prospects. New York: Wiley

[B36] Tenenbaum G. (2001). “A social-cognitive perspective of perceived exertion and exertion tolerance,” in Handbook of Sport Psychology, 2nd Edn, eds Singer R. S. Hausenblas H. A. Janelle C. M. (New York: John Wiley & Sons, Inc.), 810–820

[B37] Tun P. A. Wingfield A. (1995). Does dividing attention become harder with age – findings from the divided attention questionnaire. Aging Cogn. 2, 39–66 10.1080/13825589508256588

[B38] Utley S. Rudy M. (2006). The Art of Putting: The Revolutionary Feel-Based System for Improving Your Scores. New York: Gotham Books

[B39] Wiren G. (1991). The PGA Manual of Golf. New York: MacMillan

[B40] Young F. W. (1984). Scaling. Annu. Rev. Psychol. 35, 55–81 10.1146/annurev.ps.35.020184.000415 19368519

[B41] Zwislocki J. J. Goodman D. A. (1980). Absolute scaling of sensory magnitude: a validation. Percept. Psychophys. 28, 28–38 10.3758/BF03204312 7413407

